# Synergistic Angio-Osteogenic Effects of Copper-Releasing 3D Biocomposite Scaffolds: A Step Toward Vascularized Bone Regeneration

**DOI:** 10.21203/rs.3.rs-7159849/v1

**Published:** 2025-08-26

**Authors:** Saman Baghaei, Negar Azarpira, Maryam Paknahad, Ali Mohammad Amani, Hengameh Dortaj, Farhad Koohpeyma, Seyyed Sajad Daneshi, Ehsan Vafa, Ahmad Vaez, Fatemeh Lavaee, Lobat Tayebi

**Affiliations:** 1 Student Research Committee, School of Dentistry, Shiraz University of Medical Sciences, Shiraz, Iran; 2 Craniofacial and Cleft Research Center, Isfahan University of Medical Sciences, Isfahan, Iran; 3 Transplant Research Center, Shiraz University of Medical Sciences, Shiraz, Iran; 4 Oral and Dental Disease Research Center, Oral and Maxillofacial Radiology Department, Shiraz of Dentistry, Shiraz University of Medical Sciences, Shiraz, Iran; 5 Department of Medical Nanotechnology, School of Advanced Medical Sciences and Technologies, Shiraz University of Medical Sciences, Shiraz, Iran; 6 Department of Anatomy and Cell Biology, Mashhad University of Medical Sciences, Mashhad, Iran; 7 Endocrine and Metabolism Research Center, Shiraz University of Medical Sciences, Shiraz, Iran; 8 Stem Cells Technology Research Center, Shiraz University of Medical Sciences, Shiraz, Iran; 9 Department of Tissue Engineering and Applied Cell Sciences, School of Advanced Medical Sciences and Technologies, Shiraz University of Medical Sciences, Shiraz, Iran; 10 Biotechnology Research Center, Shiraz University of Medical Sciences, Shiraz, Iran.; 11 Oral and Dental Disease Research Center, Oral and Maxillofacial Disease Department, School of Dentistry, Shiraz University of Medical Sciences, Shiraz, Iran; 12 Institute for Engineering in Medicine, Health, & Human Performance (EnMed), Batten College of Engineering and Technology, Old Dominion University, Norfolk, VA, 23529, USA

**Keywords:** Bone regeneration, 3D-printing, Bioglass, Copper, Angiogenesis, Critical-sized defect

## Abstract

Critical-sized bone defects present significant clinical challenges due to inadequate vascularization and scaffold integration. This study developed a multifunctional 3D-printed polycaprolactone (PCL)-gelatin (Gel) scaffold reinforced with Bioglass particles (BGPs) or copper dopped BGPs (CuBGPs) to synergistically enhance angiogenesis and bone regeneration in rat model. The scaffolds were fabricated by infiltrating gelatin solutions containing BGPs or CuBGPs into the pores of 3D-printed PCL matrices, followed by freeze-drying. Comprehensive characterization of PCL-gel, PCL-gel-BGPs, and PCL-gel-CuBGPs scaffolds revealed optimal porosity (58.76±5.20, 53.27±11.83, and 54.5±7.61%), contact angle (74.53 ±6.6, 71.76±2.65, and 69.89±4.14), and controlled degradation (44.65±4.73, 47.93±2.51, and 50.58±5.68). MTT study demonstrated dose-dependent enhancement of cell proliferation, with BGPs and CuBGPs significantly improving mesenchymal stem cells (MSCs) viability by day 5. In vivo experiments in rat calvarial defects showed that Cu containing scaffolds promoted greater new bone volume compared to other groups at 12 weeks. Histological and immunohistochemical analyses confirmed robust angiogenesis and woven bone formation, with CuBGPs achieving the highest vasculature. This study provides a detailed and reproducible framework for Cu-doped scaffold fabrication, characterization, and application in critical-sized defect regeneration.

## Graphical abstract

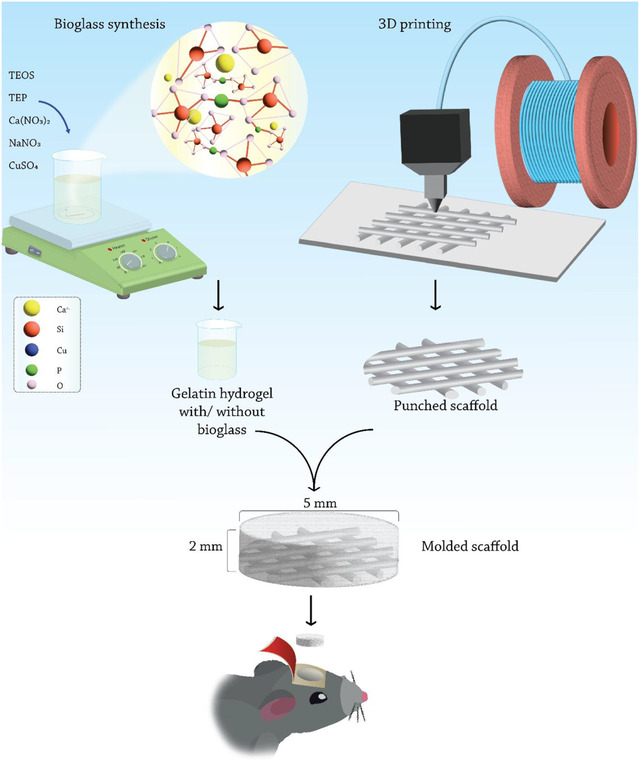

The prepared bioglass was added to the gelatin solution. Then the polymer solution was poured into the empty spaces of the printed scaffold and placed in the defect created in the rats calvaria.

## 1 Introduction

Bone tissue engineering has emerged as a transformative solution for critical-sized defects, addressing the limitations of autografts (donor scarcity) and allografts (immune rejection) [1]. However, scaffold success remains constrained by a critical challenge: inadequate vascularization, which impairs cell survival, graft integration, and functional bone regeneration—particularly in metabolically compromised defects (e.g., osteoporotic or irradiated bone) [2–4]. While 3D-printed scaffolds now achieve patient-specific geometries [5–7], their regenerative potential is often limited by material compromises between bioactivity and mechanical performance [8, 9].

To bridge this gap, composite systems combining synthetic polymers with bioactive phases have gained prominence. Polycaprolactone (PCL) provides structural integrity but suffers from poor hydrophilicity [10]; blending it with gelatin—a collagen-derived polymer rich in RGD motifs—enhances cell adhesion while preserving stability [11–13]. Bioactive glasses (e.g., 45S5) further augment functionality by releasing osteogenic ions (Ca^2+^, SiO_4_^4-^) and forming hydroxycarbonate apatite layers [14]. Strategic doping with therapeutic ions like copper (Cu^2+^) can unlock additional benefits: Cu’s pro-angiogenic effects (via HIF-1α/VEGF activation) [14], antimicrobial properties and ability to stimulate osteogenesis through BMP-2 upregulation make it ideal for vascularized bone regeneration [15–17].

In this study, we engineered a 3D-printed PCL/gelatin scaffold incorporating Cu-doped bioactive glass particles (Cu-BG, 2 mol% CuO) to synergistically promote angiogenesis and osteogenesis. Using a rat calvarial defect model—a gold standard for evaluating vascularized bone repair, we compared Cu-BG scaffolds with undoped BG controls via micro-CT, histomorphometry, and immunohistochemistry. We hypothesized that Cu^2+^ release would (1) enhance early-stage vascular infiltration and (2) accelerate defect mineralization while maintaining mechanical stability, offering a clinically translatable solution for complex bone defects.

## 2 Materials and Methods

### 2.1 Materials and reagents

All chemicals and biological materials were of analytical grade. Gelatin (type B, derived from bovine skin) and polycaprolactone (PCL; Mw = 80 kDa) were procured from Sigma-Aldrich (St. Louis, MO), along with glutaraldehyde (GA) for crosslinking. Cell culture supplements, including Dulbecco’s Modified Eagle’s Medium/Nutrient Mixture F-12 (DMEM/F12), fetal bovine serum (FBS), and penicillin-streptomycin, were obtained from Gibco (Grand Island, NY). Inorganic precursors such as tetraethyl orthosilicate (TEOS), triethyl phosphate (TEP), sodium nitrate, and calcium nitrate were supplied by Merck and the MTT assay kit was purchased from DNAbiotech, (I.R Iran). For in vivo studies, adult Sprague-Dawley rats were sourced from the Pasteur Institute (Tehran, Iran), while ketamine (Bioveta) and xylazine (Kela) served as anesthetics.

### 2.2 Synthesis of BG particles (BGPs) and Cu-doped BGPs

Copper-doped bioactive glass (CuBGPs) particles with compositions of 12% CuO (mol%) were synthesized using a sol-gel method under basic conditions, based on a standard protocol [18]. The base formulation was 45 SiO_2_–24.5 CaO–24.5 Na_2_O–6 P_2_O_5_ (mol%), with Na_2_O reduced proportionally to accommodate CuSO_4_·5H_2_O. After aging at 25 °C for 24 hours, the gel was dried at 60 °C and 120 °C, then calcined at 700 °C (heating rate: 3 °C/min) for 2 hours to form a stable glass structure. Final powders were obtained via ball milling.

### 2.3 Characterization of BGPs

Scanning electron microscopy (SEM, Quorum 150R) examined morphology and particle size, while elemental composition was confirmed using EDX (TESCAN SEM at 20 kV). Also, structural and chemical properties were analyzed using FTIR (Bruker Tensor II, Germany) and XRD (Bruker Advance D8, Germany) to assess bonding and crystallinity.

### 2.4 Scaffold fabrication

Scaffolds were fabricated using an extrusion-based 3D printer (Nika, Adli TRG, Iran) according to a published protocol [19]. Molten PCL was deposited through 300 μm nozzles to create a grid-like structure with 200 μm strut spacing in a 0°/0°/90°/90° pattern. Discs (5 mm diameter × 0.9 mm height) were printed at a speed of 5 mm/s and subsequently molded and infused with a 20% (w/v) gelatin solution containing 10% BGPs (w/w) so that final height reached 2 mm, followed by freeze-drying (Alpha 2–4 LSCbasic, Martin Christ, Germany). Structural stability and gelatin cross-linking were enhanced by immersion in a 3% (v/v) glutaraldehyde solution for 30 minutes, after which the scaffolds were rinsed three times with distilled water to remove residual reagent. A final freeze-drying step ensured complete drying and dimensional stability prior to biological testing.

### 2.5 Characterization of the scaffolds

#### 2.5.1 Surface morphology

SEM images of the 3D-printed structures were acquired. Prior to imaging, a thin layer of gold was sputtered onto the samples using a sputter coater (Q150 R, Quorum, England) to enhance surface conductivity.

#### 2.5.2 Contact angle

The wettability of scaffolds was thoroughly assessed using the sessile drop method with a static contact angle measurement system (SHARIF Solar CA500A, Iran) [18]. Measurements were taken at five distinct locations on each scaffold, and the average water contact angle was determined using distilled water (DW) [11].

#### 2.5.3 Weight loss

The degradation rate of scaffolds was determined by measuring the weight loss of the produced scaffolds in PBS solution [18]. PBS was selected due to its physiological pH of 7.4, which closely mimics the in vivo environment. In this study, the initial mass of each scaffold was recorded before submerging identical samples in 15 mL of PBS (pH 7.4) at 37 °C. At predetermined time points—days 3, 7, 14, and 30—triplicate specimens from each group were removed, dried in a vacuum oven, and reweighed. The extent of degradation was quantified as weight loss percentage using Equation 1 (Eq. 1):

(Eq. 1) $$\text{Weight loss}(\%)=\frac{W_{0}-W_{1}}{W_{0}}\times 100$$

where *W*_0_ is the initial weight of the hydrogels and scaffolds, and *W*_1_ is the dry weight after removal from PBS.

#### 2.5.4 Mechanical properties

The compressive strength of 3D-printed scaffolds (10 × 10 × 10 mm^³^) was evaluated following ASTM F2150–02 standards using a uniaxial compression tester (SANTAM STM 20, Iran) at a rate of 1 mm/min. A minimum of five samples per group were tested, with average values reported. Stress (σ) and strain (ε) were calculated as:

(Eq. 2) $$\text{Stress}(\sigma )=\frac{F}{A_{0}}$$

(Eq. 3) $$\text{Strain}(\varepsilon )=\frac{\Delta L}{L_{0}}$$

(where F = applied force, A_0_ = initial cross-sectional area, ΔL = displacement, and L_0_ = original length). The elastic modulus (E) was determined from the linear elastic region of the stress-strain curve:

(Eq. 4) $$\text{Elastic Modulus}(E)=\frac{\Delta \sigma }{\Delta \varepsilon }$$

Yield strength, defined as the onset of plastic deformation, was identified using the 0.2% offset method. The ultimate compressive strength (UCS) was recorded as the peak stress before failure, while elongation at break was measured as the percent increase in length at fracture [18].

#### 2.5.5 Porosity assessment

Drawing on established methodologies [20, 21], the porosity of the 3D-printed scaffolds was quantified using the ethanol displacement technique, as outlined in Eq. 5. The procedure involved placing a scaffold of known weight (W) into a graduated cylinder containing a measured volume of ethanol (V_1_). Following immersion, the total liquid volume was recorded as V_2_. After allowing the sample to remain in the cylinder for 10 minutes, the scaffold was removed, and the remaining ethanol volume was measured as V_3_.

(Eq. 5) $$\text{Porosity}(\%)=\frac{V_{1}-V_{3}}{V_{2}-V_{3}}\times 100$$

#### 2.5.6 Blood compatibility

The hemocompatibility of the scaffolds was assessed using Sprague Dawley rats’ blood, following the guidelines outlined in ASTM F756. Prior to testing, the blood was diluted at a ratio of 1:2.5 with normal saline and anticoagulated to prevent clotting. Each scaffold sample was incubated with 0.2 mL of the diluted blood at 37 °C for 60 minutes [18, 22]. Following incubation, samples were centrifuged at 1500 rpm for 10 minutes, with the supernatant subsequently transferred to a 96-well microplate for spectrophotometric analysis. Absorbance measurements were conducted at 545 nm using a microplate reader (POLAR Star Omega BMG Labtech, Germany). The assay included two reference controls: a positive control (0.2 mL blood in 10 mL deionized water, representing 100% hemolysis) and a negative control (0.2 mL blood in 10 mL normal saline, representing 0% hemolysis). The hemolysis percentage was calculated according to Eq. 6:

(Eq. 6) $$\text{Hemolysis}(\%)=\frac{D_{t}-D_{nc}}{D_{pc}-D_{nc}}\times 100$$

Where D_t_ is the absorbance of the sample, D_nc_ represents the absorbance value of the negative control, and D_pc_ is the absorbance value of the positive control [23].

#### 2.5.7 Cell viability test

The cytotoxic potential of the 3D-printed scaffolds was evaluated using the MTT assay with Mesenchymal stem cells (MSCs), following the established protocol [18]. Briefly, bone marrow was extracted from femurs and tibias of 6–8-week-old Sprague-Dawley rats and cultured in standard cell culture condition, including DMEM supplemented with 10% FBS and 100 U/ml penicillin, and 100 μg/ml streptomycin. For the MTT assay, 1 × 10^4^ cells/well were cultured into a 96-well plate on the sterile scaffolds. At days 1, 3, and 5, the culture media were replaced with 0.2 ml of MTT solution (0.5 mg/ml), and the plate was incubated in the dark at 37 °C for 4 hours. Following the formation of formazan crystals, the supernatant was replaced with 0.1 ml of DMSO to dissolve the crystals. Absorbance was then measured at 570 nm using a microplate reader.

### 2.6 *In vivo* study

All animal procedures were approved by the Institutional Animal Ethics Committee of Shiraz University of Medical Sciences and strictly adhered to institutional ethical guidelines for laboratory animal welfare (Approval No. IR.SUMS.AEC.1403.051). This study is reported in accordance with the ARRIVE guidelines. Forty male Sprague-Dawley rats (8–10 weeks old, 250–300 g) were randomly allocated into four experimental groups (n=5 per group per time point): (1) Sham (defect without any treatment), (2) Control (PCL-Gel), (3) PCL-Gel-BGPs (4) PCL-Gel-CuBGPs. General anesthesia was induced via intraperitoneal administration of 100 mg/kg Ketamine and 10 mg/kg Xylazine, following aseptic protocols [24]. A standardized 5 mm full-thickness critical-sized calvarial defect was created using a precision trephine drill (Dental Studio, South Korea) at 1,000 rpm under continuous saline irrigation to prevent thermal necrosis. Following scaffold implantation, the periosteal layer was repositioned and secured with 6–0 silk sutures (SUPA Medical Devices, Iran), and the skin was closed with interrupted 3–0 nylon sutures (SUPA Medical Devices, Iran).

#### 2.6.1 The cone-beam computed tomography (CBCT) scan

CBCT scans were acquired at 6- and 12-week postoperative intervals using a NewTom VGi imaging system (NewTom ORsrl, Verona, Italy) operating in high-resolution mode (0.1 mm isotropic voxel size; 6 × 6 cm field of view). To ensure assessment reliability, a blinded examiner performed quantitative analyses during two independent evaluation sessions. All image analyses were conducted under standardized viewing conditions using randomized scan sequences. Multiplanar reconstructions were generated and analyzed in three orthogonal planes (axial, coronal, and sagittal) [25].

#### 2.6.2 Tissue harvest and histological processing

Following predetermined experimental endpoints (6 and 12 weeks post-implantation), animals were humanely euthanized using an overdose of CO_2_. Calvarial specimens containing the implantation sites were immediately dissected and immersion-fixed in 10% neutral buffered formalin (pH 7.4) for 48 hours at 4°C [26]. After tissue processing and paraffin embedding, sections were prepared and stained with hematoxylin and eosin (H&E) and Masson’s trichrome (MTC). The slides were examined under a light microscope (Eclipse TS100, Nikon), and digital images were captured. Histomorphometric analysis was performed using Fiji/ImageJ (v1.54p) to identify and quantify key cell types, new and mature bones formation, and angiogenesis.

### 2.7 Statistical analysis

All data are presented as mean ± standard deviation (SD). Statistical analysis was conducted using GraphPad Prism 10, with Student’s t-test and one-way analysis of variance (ANOVA) followed by Tukey’s post hoc test for multiple comparisons. A p-value of less than 0.05 was considered statistically significant across all tests.

## 3 Results and Discussion

### 3.1 Characterization of BGPs

#### 3.1.1 Morphology, size, and elemental analysis of BGPs

**Figure 1:**
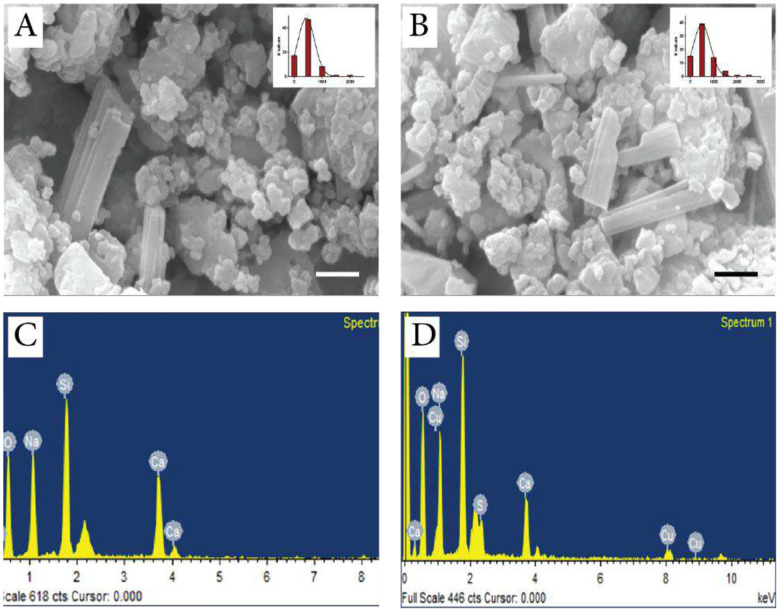
SEM micrograph of BGPs (A) and CuBGPs (B) with size distribution. Elemental analysis of BGPs (C) and CuBGPs (D); scale bar: 500 nm.

SEM was used to examine the morphology of the grounded BGPs. The analysis of SEM images revealed that the mean size of BGPs and CuBGPs were 474.7 ± 45.34 nm and 469.0 ± 36.65 nm (Figure 1A, B). Moreover, the ions within BGPs and CuBGPs were detected using EDX (Figure 1C, D).

#### 3.1.2 Chemical composition analysis of BGPs

**Figure 2:**
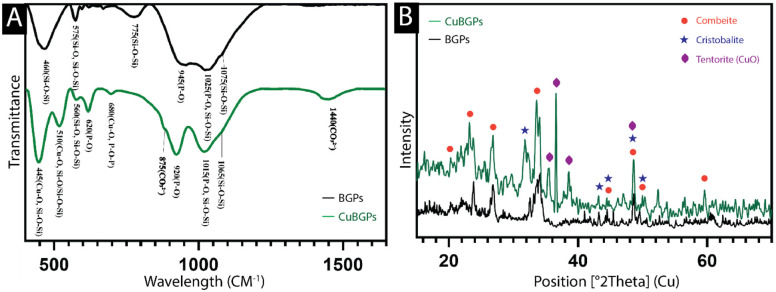
FTIR (A) and XRD (B) of BGPs and CuBGPs.

The FTIR spectrum (Figure 2A) of the BGPs and CuBGPs showed a broad stretch of the Si–O–Si (~445, ~510, ~560, ~1015, and ~1065 cm^−1^), Si–O (~510 and ~560 cm^−1^), and P–O (~620, ~920, and ~1015 cm^−1^) bands, which is characteristic of silica-based bioactive glasses. The ~445, ~510, and ~680 cm^−1^ bands also can correspond to the Cu-O. Carbonate (Ca-O) was also present with peaks at ~875 and ~1440 cm^−1^. The distinctive XRD peaks (Figure 2B) showed that BGPs had the Combeite and Cristobalite phases (JCPDS Files No. 96-900-7712 and 96-901-4260, respectively) and CuBGPs with more crystalline structure showed Combeite and Tenorite (JCPDS Files No. 96-900-7721 and 96-901-5823) [27].

### 3.2 Characterization of 3D-printed scaffolds

#### 3.2.1 The surface morphology

**Figure 3:**
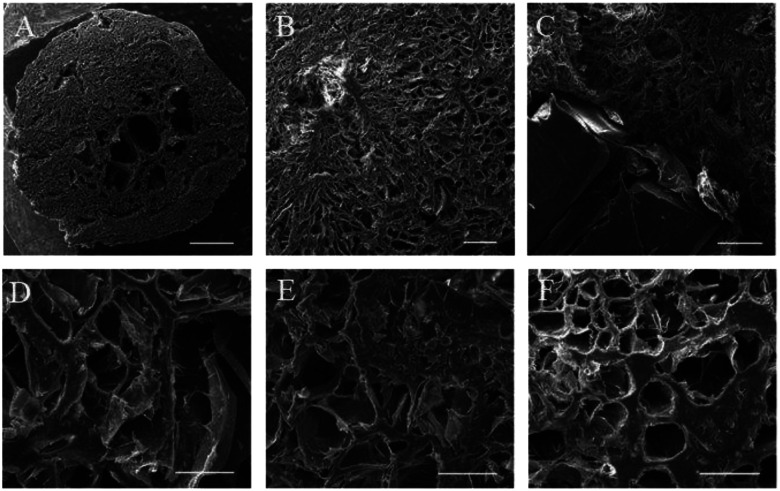
SEM images of 3D-printed scaffolds; A) The surface morphology of the 3D-printed scaffold with a 5mm diameter cast with gelation solution to reach a 2mm height, scale bar: 1mm; B) Higher magnification of the scaffold surface, scale bar: 200 μm; C) the cross-section of a scaffold showing printed PCL along with cast gelatin, scale bar: 100 μm; D-F) The surface morphology of PCL-Gel, PCL-Gel-BGPs, and PCL-Gel-CuBGPs scaffolds, scale bar: 100 μm.

The analysis of SEM images with ImageJ showed that all scaffolds had the same cancellated morphology with irregular-shaped pores with approximate mean diameters of around 50–100 μm (Figure 3). According to previous studies, the minimum required pore size for almost all tissue engineering scaffolds is considered to be around 50 μm [28, 29]. The small pores are suited for single-cell homing, and the larger ones are appropriate for vascular and soft tissue invasion. When osteoblasts were grown in scaffolds with varying pore sizes, it was found that they populated the smaller holes (40 μm) more densely but that the larger pores (100 μm) aided in cell migration. However, the varying pore diameters unaffected mineralization depth and cell penetration [28–30]. As a result, all our scaffolds could meet the minimum required pore size and were suitable for bone tissue engineering.

#### 3.2.2 Contact angle measurement

**Figure 4:**
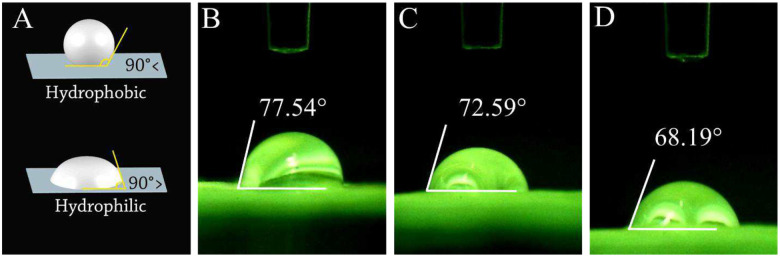
The contact angle measurements of different scaffolds; A) a schematic illustration showing the hydrophobic and hydrophilic natures of biomaterials measured by the sessile drop method; b-d) contact angle values of PCL-Gel, PCL-Gel-BGPs, PCL-Gel-CuBGPs scaffolds, respectively.

Previous studies showed that a water contact angle below than 90°, especially ranging from 40° to 70° is suitable for cells to attach and grow [31]. The contact angle amount is related to different parameters, such as the ingredients’ nature and surface properties [32]. In this study, we used 3D-printed PCL with hydrophobic and gelatin with a hydrophilic nature; therefore, the final result is expected to be adjusted. On the other hand, cross-linking the scaffolds can also change the contact angle of materials. As can be seen in Figure 4, the contact angle values for the PCL-Gel, PCL-Gel-BGPs, and PCL-Gel-CuBGPs scaffolds were measured to be 74.53 ±6.6, 71.76±2.65, and 69.89±4.14, respectively. These results showed that all scaffolds had hydrophilic properties and were suitable for cell attachment.

#### 3.2.3 Weight loss

**Figure 5:**
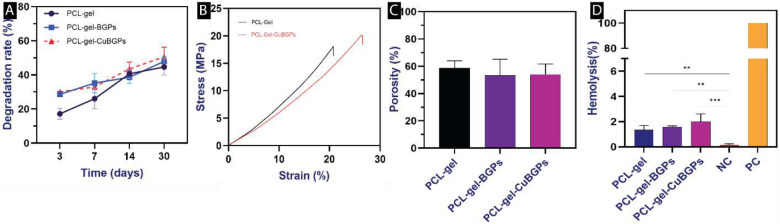
A) Histogram comparing the prepared scaffolds’ weight-loss percentages at 3, 7, 14, and 30 days, n = 3; B) Representative stress-strain curves upon compression testing of PCL-Gel and PCL-Gel-CuBGPs scaffolds; C) Porosity assessment, n=5; and D) Hemocompatibility test showed all scaffolds were non-hemolytic, n=6. **p<0.01, ***p<0.001.

The degradation study of PCL-gel, PCL-gel-BGPs, and PCL-gel-CuBGPs over a period of 30 days reveals distinct trends in their degradation rates (44.65±4.73, 47.93±2.51, and 50.58±5.68, respectively). However, the rate of degradation varies among materials (Figure 5A). This suggests that the incorporation of BGPs and CuBGPs enhances the degradation kinetics compared to pure PCL-gel, which can be attributed to the presence of bioglass particles, which are known to promote hydrolytic degradation through increased surface area and catalytic activity. It seems that BGPs and CuBGPs introduce additional sites for water penetration and chemical reactions, accelerating the breakdown of the polymer matrix [33].

#### 3.2.4 Mechanical property

The compression test results revealed distinct mechanical behaviors between PCL-Gel and PCL-Gel-CuBGPs (Figure 5B, Table 1). PCL-Gel exhibited lower stress values under increasing strain. The improved compressive strength of PCL-Gel-BGPs can be attributed to the reinforcing effect of the bioglass particles, which likely enhanced the structural integrity of the composite material. The bioglass particles may have facilitated better load distribution within the matrix, thereby increasing its ability to withstand compressive forces [34]. These findings align with previous studies highlighting the role of inorganic fillers in polymer composites for mechanical reinforcement [18, 35].

#### 3.2.5 Porosity measurement

The porosity results for PCL-gel-BGPs indicate a decreasing trend in porosity as the fraction of Bioglass particles increases. The porosity percentages were measured to be about 58.76±5.20, 53.27±11.83, and 54.5±7.61 for PCL-Gel, PCL-Gel-BGPs and PCL-Gel-CuBGPs scaffolds (Figure 5C). Considering that a part of these constructs was relatively thick PCL 3D-printed struts, which causes a decrease in the final porosity percentage. Higher concentrations of Bioglass particles contribute to a denser, less porous structure. This reduction in porosity may be attributed to the filling effect of the Bioglass particles, which occupy spaces within the PCL-gel matrix, thereby decreasing void formation. Lower porosity values reflect a highly compacted structure, which may enhance mechanical properties like compressive strength but could potentially limit cell infiltration and nutrient diffusion [36].

#### 3.2.6 Hemocompatibility test

Another essential quality of implantable constructs is their hemocompatibility, which determines the scaffold’s compatibility with the patient’s blood cells. According to the ASTM F756–08 standard, a hemolytic percentage below 2%, between 2–5%, and above 5% is considered non-hemolytic, slightly hemolytic, and hemolytic. The results indicated that the percentage of hemolysis in the produced scaffolds was much lower than in the positive control (p<0.0001). The findings showed that the PCL-Gel, PCL-Gel-BGPs, and PCL-Gel-CuBGPs scaffolds were all non-hemolytic (Figure 5D).

#### 3.2.7 Cytocompatibility test

**Figure 6:**
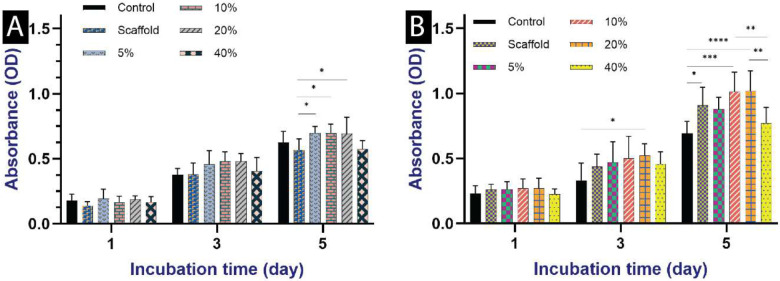
Viability and proliferative activity of MSCs cultured on the fabricated scaffolds containing BGPs (A) and CuBGPs (B), as determined by MTT assay, n=8. *p < 0.05, **p < 0.01, ***p < 0.001, and ****: p < 0.0001.

The experimental results revealed a significant enhancement in cellular proliferation on PCL-Gel scaffolds containing 20% CuBGPs compared to other experimental groups at the 3-day post-seeding interval (Figure 6). By day 5, the 20% CuBGPs group continued to exhibit superior proliferative activity, further corroborating the beneficial role of Cu incorporation. Quantitative cell proliferation assays confirmed these observations, demonstrating that the inclusion of Cu within the scaffold architecture not only elicited no cytotoxic effects but also markedly stimulated cellular expansion within the first five days of culture. These findings underscore the optimal cytocompatibility of the Cu concentrations employed in this study.

Biocompatibility remains a critical determinant in tissue engineering, particularly for in vivo defect regeneration. A pivotal consideration in our investigation was the precise modulation of Cu^2+^ ion concentration to balance bioactivity and cytotoxicity. While excessive Cu^2+^ ions can induce oxidative stress-mediated cytotoxicity, our study delineates an optimized concentration range that maximizes therapeutic efficacy while minimizing adverse cellular responses [37]. Notably, prior research has demonstrated that 1 mmol/L Cu exerts robust antibacterial effects while maintaining minimal cytotoxicity in vitro [38]. Furthermore, the incorporation of 3 mol% CuO into polymeric scaffolds has been shown to enhance cellular proliferation and viability [35]. However, exceeding this threshold can impede cell growth, as excessive Cu may trigger cytotoxic effects, including an initial burst release that disrupts cell adhesion and proliferation [39]. Based on the quantitative assessment of cellular metabolic activity via the MTT assay, PCL-Gel scaffolds incorporating either 20% BGPs or 20% CuBGPs were identified as the optimal compositions and thus selected for all experimental evaluations in this study.

### 3.3 In vivo study

#### 3.3.1 CBCT scan

**Figure 7:**
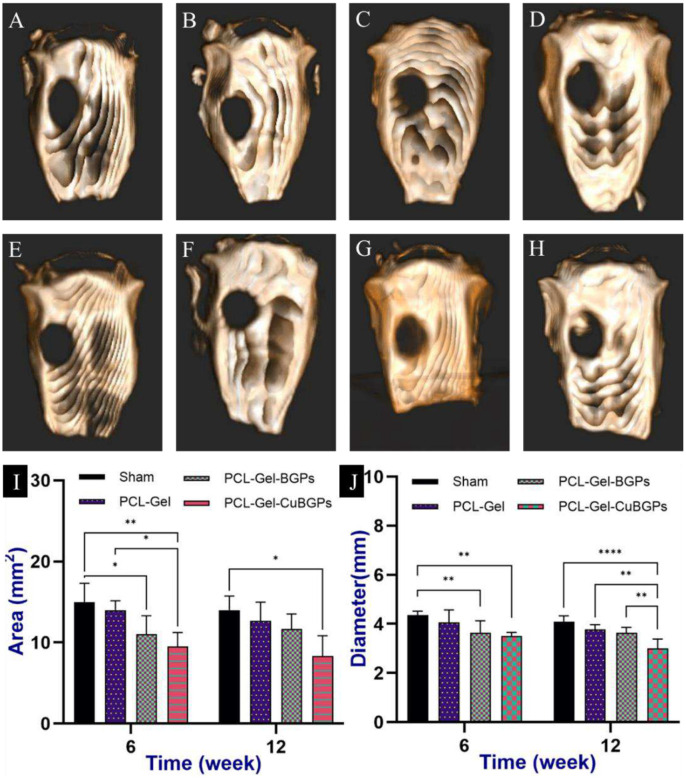
Representative CBCT images illustrate bone regeneration across experimental groups at two critical time points: (A-D) 6 weeks and (E-H) 12 weeks post-surgery. Quantitative analysis includes (I) defect area measurements and (J) the average of maximum and minimum defect radii, comparing outcomes between groups over the 6- and 12-week implantation periods.

To assess bone regeneration within the defects, CT imaging—the gold standard for bone evaluation—was employed. CBCT was done at 6 weeks (Figure 7A–D) and 12 weeks post operation (Figure 7E–H). In the sham and control groups, no significant new bone formation was observed in the fracture site six weeks post-surgery. Interestingly, scaffolds incorporating BGPs showed a reduction in empty defect area compared to the sham and control groups at this early stage. Yet, the most promising outcomes were seen with CuBGPs-loaded scaffolds. By the 12-week mark, only CuBGPs-containing scaffolds demonstrated a substantial reduction in defect area relative to the sham group (Figure 7I). When analyzing defect diameter, all BGP- and CuBGP-infused scaffolds exhibited a notable decrease compared to controls at six weeks. However, after 12 weeks, CuBGPs scaffolds outperformed the rest, showcasing the most effective defect closure (Figure 7J).

#### 3.3.2 Histopathology findings

**Figure 8:**
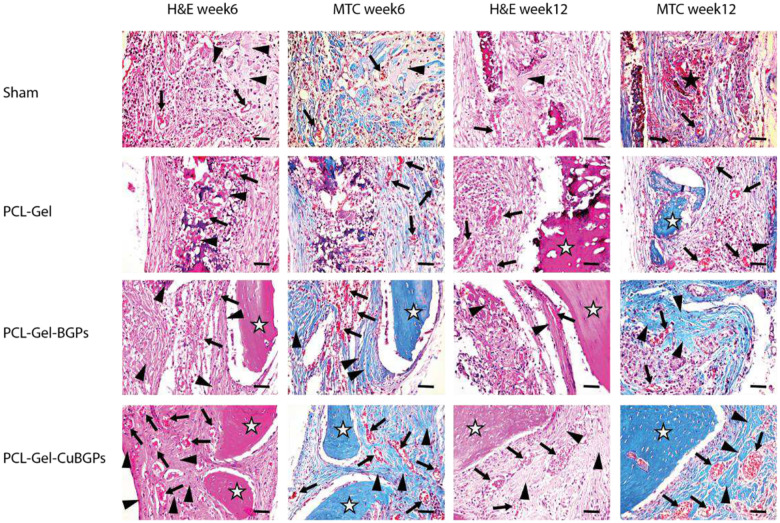
Histomorphological evaluation of bone regeneration in rat calvarial defects treated with various scaffolds at 6- and 12-week intervals. Representative H&E and MTC stained sections demonstrate distinct tissue responses: LACT: black star, NB: arrowhead, MB: white star, and angiogenesis: arrow. Scale bar: 100μm.

Histomorphometric analysis via H&E and MTC staining revealed distinct tissue responses among experimental groups. While control and sham groups exhibited defective healing characterized by loose areolar connective tissue (LACT) infiltration—comprising disorganized collagen fibers and immature vasculature—scaffolds incorporating BGPs and CuBGPs demonstrated superior osteoconductive potential. Notably, PCL-Gel-BGPs and PCL-Gel-CuBGPs constructs facilitated robust new bone formation, evidenced by the presence of mature bone (MB) and woven bone (WB). Defects treated with scaffolds, including CuBGPs, induced significant angiogenesis, with well-developed vascular networks (Figure 8).

From a biomaterials perspective, copper oxide (CuO), a Cu-component in our CuBGPs, incorporation offers distinct advantages for tissue engineering [40, 41]. Compared to alternative metallic dopants, CuO demonstrates superior chemical stability, and polymer compatibility while enhancing key scaffold characteristics—including mechanical integrity, porosity, and degradation kinetics [40, 42]. These attributes, combined with copper’s inherent antibacterial properties, address multiple challenges in bone tissue engineering by simultaneously supporting osteogenesis while mitigating infection risks [43, 44].

As an essential trace element, copper exerts its osteogenic effects primarily through three synergistic mechanisms: serving as a catalytic cofactor for lysyl oxidase-mediated collagen crosslinking and cytochrome c oxidase-dependent mitochondrial respiration, activating osteogenic differentiation via Akt/Runx2 signaling cascades, and promoting angiogenesis through HIF-1α/VEGF pathway stimulation [45]. This concentration-dependent bioactivity is particularly evident in vascular development, where optimized copper release significantly enhances both capillary density and maturation [46, 47]. The coppers’ ability to stimulate osteoblast proliferation and alkaline phosphatase activity while maintaining pro-osteogenic gene expression through Von Hippel-Lindau (VHL)-mediated HIF-1α stabilization [48].

Furthermore, copper supplementation addresses deficiency-related pathologies that compromise collagen integrity and bone strength [16, 49]. What emerges from these observations is copper’s unique capacity to simultaneously coordinate both the structural organization of bone matrix and the cellular differentiation processes essential for osseous repair [50]. This dual functionality, combined with its dose-dependent angiogenic properties, establishes copper-doped scaffolds as particularly effective platforms for bone tissue engineering, capable of addressing multiple challenges in skeletal regeneration through a single therapeutic agent.

## 4 Conclusion

The regeneration of critical-sized bone defects demands scaffolds that simultaneously address osteogenesis, angiogenesis, and microbial resistance—a triad seldom achieved by conventional materials. In this study, we engineered 3D-printed PCL-gelatin scaffolds functionalized with Cu-doped bioactive glass particles (CuBGPs) to meet this challenge. The composite design leveraged the mechanical stability of PCL, the cell-adhesive properties of gelatin, and the pro-regenerative effects of CuBGPs, which collectively enhanced pore architecture, hydrophilicity, and degradation kinetics. *In vitro*, the scaffolds supported osteoblast proliferation and extracellular matrix deposition, while *in vivo* implantation in rat calvarial defects stimulated significant vascular infiltration and new bone formation over 12 weeks, as evidenced by CBCT and histomorphometric analysis. Unlike single-phase scaffolds, our composite system balances structural integrity with bioactivity, offering a scalable solution for defects where vascular supply is limiting. These findings underscore the potential of ion-doped composite scaffolds to bridge the gap between *structural* and *functional* bone regeneration.

## Figures and Tables

**Table 1.** Mechanical properties of 3D-printed scaffolds.

	Compression module (MPa)	UCS (MPa)	Axial Shortening (%)	Yield strength (MPa)
PCL-Gel	1.59±0.16*	18.22±1.49	−19.91±2.08***	10.09±2.04
PCL-Gel-CuBGPs	1.84±0.17*	20.24±2.45	−27.83±1.84***	10.25±2.19

* *p* < 0.05;

*** *p* < 0.001, n = 5. UCS: Ultimate Compressive Strength.

## Data Availability

The datasets used and/or analyzed during the current study available from the corresponding author on reasonable request.
